# Retraction: Heidari, A.; Vedad, S.; Heidari, N.; Ghorbani, M. Flux Dynamics in Y358 Superconductors. *Materials* 2012, *5*, 882-888.

**DOI:** 10.3390/ma5122816

**Published:** 2012-12-12

**Authors:** Shu-Kun Lin

**Affiliations:** MDPI AG, Kandererstrasse 25, CH-4057 Basel, Switzerland; E-Mail: lin@mdpi.com

It has been brought to our attention by a reader of *Materials* that substantial portions of this article [1] have been translated from another publication [2] without credit. After confirming this to be the case with the authors, we have determined that indeed this manuscript clearly violates our policy of originality of all material submitted for publication and the generally accepted ethics of scientific publication. Consequently, the Editorial Team and Publisher have determined that it should be retracted. We apologize for any inconvenience this may cause.
